# MALDI Mass Spectrometry on High-Density Droplet Arrays: Matrix Deposition, Selective Removal, and Recrystallization

**DOI:** 10.1021/acsmeasuresciau.4c00016

**Published:** 2024-07-05

**Authors:** Simon F. Berlanda, Maximilian Breitfeld, Petra S. Dittrich

**Affiliations:** Department of Biosystems Science and Engineering, ETH Zurich, Basel CH-4056, Switzerland

**Keywords:** MALDI mass spectrometry, droplet array, sample preparation, sublimation, droplet microfluidics

## Abstract

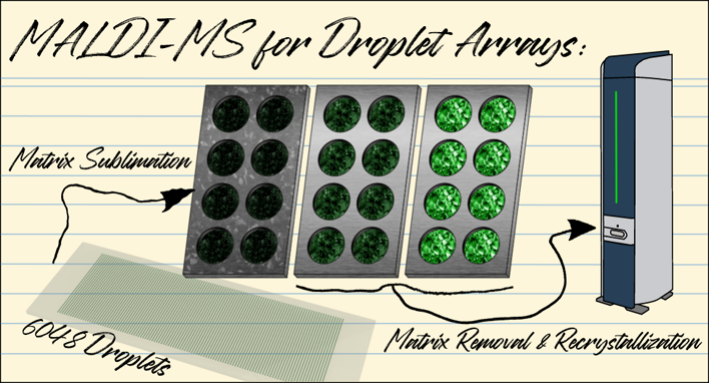

High-density droplet arrays are emerging as a powerful tool for high-throughput bioanalytical applications. These arrays are formed of thousands of nanoliter droplets, which can be analyzed by various optical and spectroscopic methods as well as label-free matrix-assisted laser desorption/ionization mass spectrometry (MALDI-MS). However, special precautions are required for the massive number of small droplets, particularly in the deposition of matrix compounds. Here, we introduce a new workflow for the analytical preparation of an array comprising 6048 droplets, which significantly improves the intensity of the MALDI-MS signals. We deposited matrix compounds in a custom-made sublimation chamber followed by a recrystallization step to achieve significant signal intensity increases for three model proteins with low, medium, and large masses, respectively. Furthermore, selective removal of the matrix before recrystallization enhanced the spatial resolution and increased the signal intensity by an average of 57%. This method can be easily standardized and upscaled for the preparation of an even larger number of droplets per array for MS analysis.

## Introduction

Droplet microfluidics is a versatile technique for high-throughput bioanalytical assays such as in single-cell sequencing^1^ or directed evolution.^2^ Monodisperse droplets in the range of nanoliter or picoliter volumes are continuously created on a microfluidic device by injecting the aqueous sample into a stream of a water-immiscible fluid. Droplet manipulation tools, such as pico-injection, droplet fusion, or splitting and sorting, are available to perform subsequent assay steps. In standard droplet microfluidics, the samples are encapsulated and guided through a microchannel. While this provides excellent throughput due to the continuous flow, the droplets remain hardly accessible for further recovery or more detailed analysis.

An alternative method is the formation of open droplet arrays, where droplets are settled on a surface at sites, which are either defined by enhanced wettability, by flat walls surrounding the droplet hosting site, or by both. Droplet arrays provide spatially separated and individually accessible samples in nanoliter droplets, which is beneficial for long-term biological assays, e.g., for monitoring of cells in a specified droplet over time.^3−5^ The droplets can be preproduced in microchannels and then distributed onto the substrate.^3^ Furthermore, electric field-based techniques leverage electrowetting-on-dielectric principles to manipulate droplets on digital microfluidic platforms, allowing for dynamic reconfiguration of droplet arrays.^6,7^ As a less complex alternative, droplet formation can occur on a surface with a pattern of strongly hydrophobic and hydrophilic areas. An aqueous solution, dropped onto the substrate, spreads across the strongly hydrophilic areas and is excluded from the hydrophobic areas, thereby forming droplet arrays.^8,9^

Droplet arrays are mainly analyzed by optical methods, such as fluorescence microscopy. While being sensitive and fast, fluorescence-based methods require specific labels which can limit the scope of analysis and potentially introduce unwanted changes in reactants upon binding. To broaden the analytical scope, alternative methods have been employed in recent years such as absorption spectroscopy,^10^ Raman spectroscopy,^8,11^ and mass spectrometry.^12^ Analysis with mass spectrometry (MS) confers significant advantages, most importantly, the direct identification and structural analysis of compounds. Previous studies carried out with droplet arrays illustrated the utilization of matrix-assisted laser desorption/ionization (MALDI)-MS for a wide range of targets ranging from small molecules to proteins.^13−16^ This hyphenation of droplet arrays and MS opens new avenues for studying biological systems, for investigating cell-produced compounds and enzymes,^3^ for determining the effects of drugs on cells,^16^ or for profiling metabolic heterogeneities in cell populations.^17^ For any typical MALDI-MS workflow, analyte ionization requires the deposition of a high laser energy-absorbing matrix.^18^ Numerous studies have shown that matrix preparation and deposition are crucial for ionization efficiency and shot-to-shot reproducibility.^19^ Standard methods of matrix delivery include manual pipetting of a matrix solution, precoating, or spray coating a matrix onto a steel target plate. These established methods are not suitable for high-density droplet arrays. For the deposition of a matrix compound onto droplet arrays, where the droplet volume and the distance between droplets are very small, special care and optimization are necessary to obtain a homogeneous matrix layer over all droplets. In addition, when the matrix compound covers the array with a liquid film, analyte diffusion between droplets may be facilitated and thereby could lead to cross contamination compromising the spatial isolation of droplets in the arrays. Sublimation-based matrix deposition offers a liquid-free matrix transfer with the added benefit of matrix purification during the process.^20^ Sublimation can be employed for many common matrix compounds such as 2,5-dihydroxybenzoic acid (2,5-DHB) and sinapinic acid that do not decompose under reduced pressure and high temperature.^37^ Furthermore, the sample and matrix are well homogenized for an efficient energy transfer, and sample diffusion outside of the wells is mitigated. Recrystallization was reported as beneficial for sample-matrix integration in tissue slices^21^ and standard target plates,^22^ but so far this workflow has not been adapted for droplet arrays.

The preparation of MALDI-MS samples becomes notably challenging when dealing with droplet arrays, primarily due to their high density levels, small volumes, and typically low analyte levels. The inclusion of oil and surfactants in the array generation process significantly influences sample drying behavior, a phenomenon accentuated by the presence of inorganic salt concentrations.^13^ Inhomogeneous matrix deposition can additionally distort the readout of the array. In addition, wall effects and convection during drying can lead to precipitation of analyte from the middle of the wells toward the edges.^23^ To address this issue, attempts have been made to minimize these factors by redesigning flat wells into concave microbowls, which requires a more complex manufacturing process.^24^

Here, we use a sublimation method for the application of matrix compounds on high-density open droplet arrays, which improves measurement sensitivity, is easy to implement, and is straightforward to upscale. We tested this method with three biomolecules covering a wide mass range, where the signal intensity significantly increased after the vacuum-sublimated matrix deposition for each biomolecule. Furthermore, when removing the matrix around the droplets followed by a recrystallization step, the signal retained the strong spatial distribution of the initial droplet array, especially when compared to the same process without this step.

## Experimental Section

### Chemicals

2,5-Dihydroxybenzoic acid (2,5-DHB), methanol, insulin from bovine pancreas, FITC-labeled recombinant insulin, and bovine serum albumin (BSA) were purchased from Sigma-Aldrich (St. Lois, USA). Angiotensin II was ordered from RayBiotech (Georgia, USA). HFE-7500 oil was acquired from Fluorochem (Hadfield, UK). The epoxy-based photoresist SU-8 3025 and isopropyl alcohol were purchased from Micro Chemicals (Ulm, Germany). All chemicals were obtained with the highest available purity and used without further purification steps.

### Fabrication of Plates

The high-density array plates with several thousands of wells (diameter: 250 μm) were fabricated in the cleanroom using photolithography. The bottom surface of the wells was hydrophilic and surrounded by a hydrophobic photoresist layer. First, a 300 nm indium tin oxide (ITO)-coated borosilicate wafer (Siegert Wafer, Aachen, Germany) was dehydrated at 200 °C for 5 min. After 3 min of plasma activation (Tepla Gigabatch, Wettenberg, Germany), a 20 μm SU-8 layer was spin-coated at 5000 rpm for 45 s. Afterward, the wafer was soft-baked for 1 min at 65 °C followed by another 10 min at 95 °C. The wafer was covered by a photolithography mask and exposed to UV light to transfer the design onto the wafer. After postbaking for 1 min at 65 °C and 5 min at 95 °C, the wafer was developed with Mr. Dev 600 (Micro resist technology, Berlin, Germany) for 5 min to remove the unpolymerized SU-8. Afterward, the wafer was cleaned with isopropyl alcohol and deionized water and hard-baked overnight with a slow temperature ramp up to 180 °C. Finally, the wafer was cut to obtain high-density array plates with standard microscopy-slide dimensions.

### Preparation of High-Density Droplet Arrays

**Figure 1:**
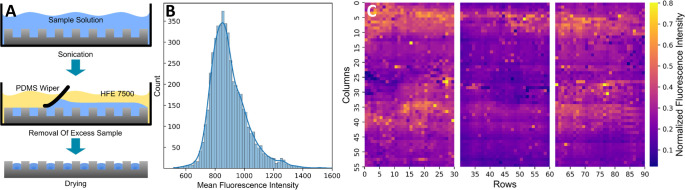
Droplet array generation and characterization. [A] Workflow for the generation of open droplet arrays on ITO-coated plates (not to scale). [B] Distribution of the fluorescence intensity of 40 μM FITC-tagged insulin on one plate. Each data point is comprised of the mean fluorescence intensity over one well (*n* = 4950 sample wells). [C] Fluorescence heatmap of an array plate filled with 40 μM FITC-tagged insulin. Each pixel represents the normalized intensity of one individual well indicating the filling level with sample (*n* = 4950 sample wells).

The ITO plate with patterned SU-8 wells was dipped in the sample solution. After 2 min of sonication at 45 kHz, the plate was subsequently transferred into HFE 7500 oil, where the individual wells were isolated through the removal of the excess sample with a polydimethylsiloxane (PDMS) wiper (Figure 1A). At the end of this simple process, the plate was air-dried.

### Optical Imaging

Fluorescence imaging of the 40 μM FITC-labeled insulin solution was carried out on a Nikon Eclipse Ti2 at an excitation wavelength between 446 and 486 nm and an emission wavelength between 500 and 550 nm. Data analysis was performed with a custom script (Matlab) that extracted the mean intensity values over the whole area of each predefined well. The initial bright-field imaging was performed on a Nikon Eclipse TS100.

### MALDI Matrix Deposition

150 mg of the 2,5-DHB matrix in acetone was added to the bottom of a custom-made sublimation chamber (Figure S1).^21,25^ The plate was attached to the bottom of a coldfinger and inserted into the chamber. The chamber was evacuated to a pressure of 0.2 mbar by using a rotary vane pump. An ice slush was next filled into the coldfinger, and the system was allowed to thermally stabilize for 3 min. Afterward, a copper heating bath was used to heat the chamber to 140 °C for 15 min to sublime the 2,5-DHB matrix.

The comparative matrix deposition method was performed with an ImagePrep system from Bruker. We used the following parameters for automatic mode, according to the supplier’srecommendation: 0.8–1 s of spray, 10 s incubation, and 60 s of drying for a total of 50 spray cycles.

### Selective Matrix Removal and Recrystallization

Matrix surrounding the wells was removed by placing adhesive tape on the sublimated slide. The tape was gently pressed down and peeled off in one swift motion. During this procedure, the matrix remains in the wells while it is removed from the surrounding hydrophobic SU-8 layer. Recrystallization was performed in a saturated H_2_O:MeOH (1000:5 ratio, v/v) atmosphere at 60 °C for 2.5 min.^26^ Finally, the slide was dried under a N_2_ stream.

### MALDI-MS Measurements

Analysis was carried out with a RapifleX MALDI Tissuetyper (Bruker Daltonics). 500 shots per well were collected with random laser movement, where the laser was moved after accumulating 100 shots at the same position. All resulting spectra were summed to represent one well. The laser power was fixed at 80% with a repetition rate of 10 kHz. A mass range of *m*/*z* 900–6000 was chosen with a digitization rate of 1.25 GS/s for angiotensin II and bovine insulin in positive reflector TOF/TOF mode. Bovine serum albumin was measured in linear TOF mode at a mass range of *m*/*z* 60 000–70 000. Spectra were accumulated by FlexControl software (v.4.0 Build 46, Bruker) with a custom target geometry and exported using FlexAnalysis Batch Process software (v.4.0 Build 14). Image-guided MS was carried out using flexImaging (v.5.0 Build 89, Bruker) with a stitched micrograph of the array plate at 50 μm × 50 μm laser raster size with the same detector settings as the well measurements described above.

### MALDI-MS Data Analysis

We used Python (v.3.10.2, Python Software Foundation, Wilmington, USA) for data processing.^27^ The signal-to-noise threshold was set to 10 with a 0.002 peak binning tolerance. The signal intensity of the most abundant mass peak at *m*/*z* 1046 for angiotensin II, *m*/*z* 5733 for bovine insulin, and *m*/*z* 66620 for bovine serum albumin, respectively, was taken for comparison. The imaging runs were evaluated using flexImaging from Bruker. Statistical analysis was performed using the Mann–Whitney U test, and significance was defined as *p* < 0.05(*), *p* < 0.01 (**), and *p* < 0.001 (***).

## Results and Discussion

### Creation of the Droplet Array

We created ultrahigh-density droplet arrays with the goal of imaging them with MALDI-MS, which requires fast sample preparation (i.e., the generation of the droplet array) and homogeneous matrix deposition. Therefore, we first adopted and improved a simple and rapid method to generate high-density droplet arrays.^8^ We manufactured array plates by photolithography with either (a single block of) 6048 wells or 3 blocks with 4950 wells in total. Each plate comprised 250 μm diameter hydrophilic wells, surrounded by a continuous 20 μm high hydrophobic photoresist layer. With this method, the amount, distance, and geometry of the wells can easily be adapted according to the requirements of the application. To rapidly fill the wells, the plate was dipped into the analyte solution and sonicated to remove the bubbles. Afterward, the plate was placed in a bath of perfluorinated oil, where the individual wells were isolated through the removal of excess samples (Figure 1A). The droplet array, comprising droplets with a volume of 1 nL, was generated within a few minutes and without any specialized instruments. Of note is that we did not use any surfactant in the oil to avoid possible analyte diffusion through micelle formation,^28,29^ cross talk^30^ between wells, and potentially detrimental effects on the ionization.^31^

We assessed the homogeneity of droplet deposition by using fluorescently labeled insulin dissolved in the analyte solution. Image analysis revealed that each well contained an analyte solution (mass spectra in Figure S2). The fluorescence intensity had a mean of 896 ± 127, which is a satisfying small distribution (Figure 1B,C). Small deviations in the filling height and optical artifacts contribute to the variations of the fluorescence signal, e.g., scattered light at the interface of the hydrophilic wells to the hydrophobic coating (Figure S3). To study potential cross talk between the wells, we used a previously reported droplet spotting platform to deposit alternating rows of analyte and water blanks (Figure S4).^3^ It could be confirmed that the analytes are not diffusing to adjacent droplets when the droplets are covered by oil (Figure S5).

### Crystallization after Matrix Application

**Figure 2:**
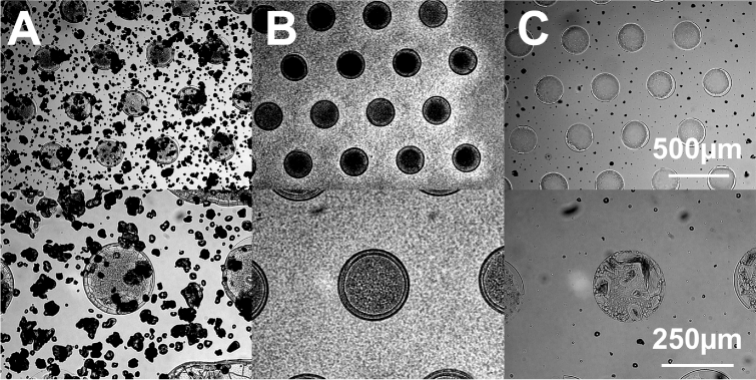
Micrographs of different matrix deposition methods on the same droplet plate with 40 μM bovine insulin and 2,5-DHB as a matrix. [A] Matrix deposition through a standard matrix sprayer. [B] Vacuum-sublimated matrix. [C] Recrystallized vacuum-sublimated matrix.

Next, we prepared the array for MALDI-MS measurements and applied the matrix compound here 2,5-dihydroxybenzoic acid (2,5-DHB). The method of matrix application is known to play an important role in crystal size and morphology.^32,33^ The commonly used method of spray-based deposition often results in large crystal sizes, although improvements have been reported recently.^32^ Indeed, in our system, large, unevenly distributed crystals formed after spray deposition on the plate (Figure 2A). In contrast, sublimation can be exploited to deposit a suitable matrix, which also further purifies it during the process.^34^ Here, we used a custom-made chamber (Figure S1) to sublimate matrix compounds for deposition on up to two droplet arrays simultaneously. As can be seen in the micrographs, this procedure led to an evenly coated plate with fine crystals (Figure 2B). In the case of sublimation, the matrix is layered on top of the sample without mixing. As such, a laser beam will only affect the uppermost layer,^34^ and a high number of laser shots on the same spot are necessary to ablate enough sample and matrix^35^ for sufficient signal. In consequence, the signal intensity is low, as described below and later in Figure 4. It is generally well-known that a homogeneous mixing of sample and matrix in a defined ratio improves efficiency for primary and postionization from the MALDI plume.^36^

Matrix recrystallization in tissue slices has been reported to improve the signal yield for various analytes^21,37^ but has not been applied so far in high-density droplet arrays. This simple process consists of transferring the plate into a humidified atmosphere for several minutes, allowing the reliquefaction and mixing of the matrix with the sample. Furthermore, this step also reduces the uneven distribution of the sample after drying. Evaporation-driven capillary flow results in a higher concentration of sample toward the frontier of the wells, commonly known as the coffee-ring effect.^38^ When we performed this additional step, the sublimated matrix did not evenly spread out over the surface but crystallization was mainly observed within the wells, with a few smaller crystals still visible across the entire plate (Figure 2C). We assume that the difference in surface wetting led to this effect: crystal seeds form preferentially in the hydrophilic wells and the growing crystal stays within these wells, while the hydrophobic surface coating inhibits the formation and growth of crystals.^23^

**Figure 3:**
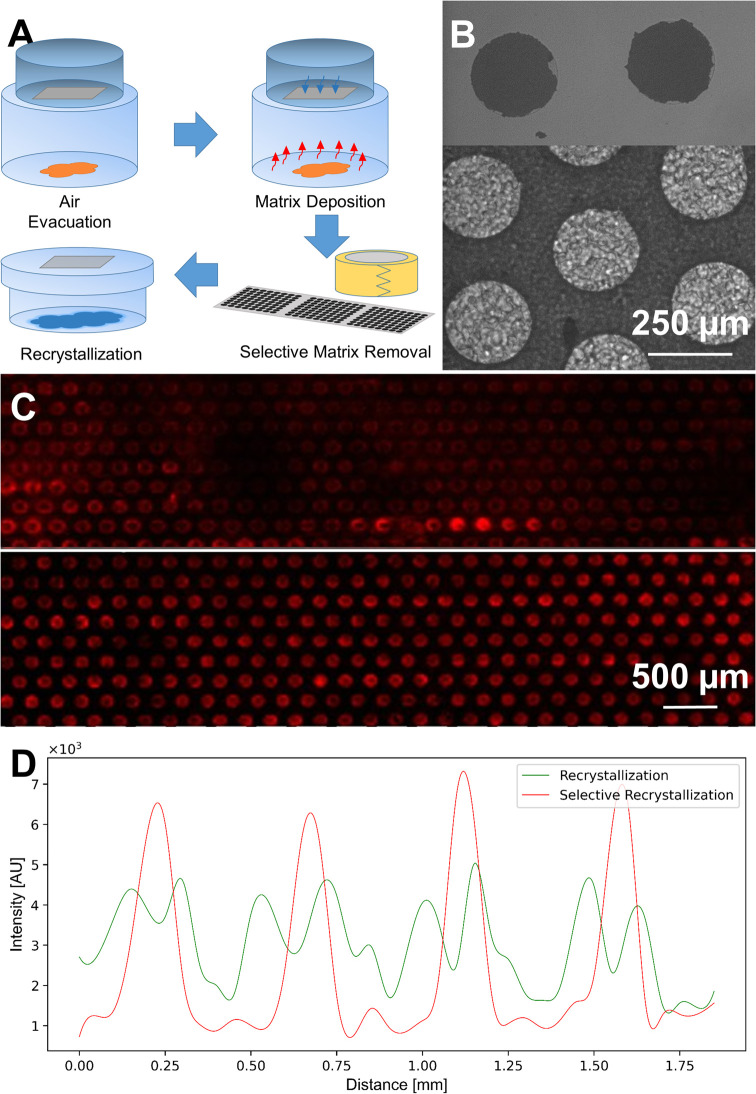
Sample preparation process and effect of matrix removal on the signal intensity. [A] Optimized sample preparation, which includes matrix sublimation, removal of the matrix between the wells, and a recrystallization step. [B] Top: microarray wells after matrix removal. The deposited matrix surrounding the wells was removed. Bottom: adhesive tape after matrix removal had the opposite pattern. The matrix compounds appear in dark gray, and the wells without adherent matrix compounds appear in light gray. [C] Image-guided MALDI-MS map comparing the absolute intensity of 40 μM bovine insulin after recrystallization without (top) and with (bottom) selective removal of the matrix. [D] Absolute signal intensity depicting the distribution of the sample and matrix across four wells (with a laser focus of 50 μm).

Matrix recrystallization involves reliquefaction, during which the analyte can diffuse out of the wells into the liquid matrix. To prevent this loss of analytes and remove the residual small crystals outside the wells, we removed the matrix before recrystallization simply by using adhesive tape (Figure 3A). The tape was applied across the sublimated slide, carefully flattened down, and then peeled off in one motion. This process removes the loosely attached matrix around the wells, while the wells are still filled with matrix (Figure 3B). For visualization, untagged bovine insulin was deposited on the plate. Mass spectrometry imaging was conducted after matrix deposition by sublimation and recrystallization with and without the matrix removal step (Figure 3C). As is visible in the images, the matrix removal led to clearly defined wells and an increase in the signal intensity of the analyte. We further imaged the signal intensity distribution across the wells. Without the matrix removal but before recrystallization, analytes were preferentially detected near the edges of the wells (Figure 3D). Drying droplets experience an outward, radial flow resulting in a maximum evaporation flux at the edge of the droplets.^39^ This ring-like residue was caused by a coffee-ring effect, leading to an outward concentration gradient.^38^ By removing the matrix around the well before recrystallization, the hydrophobic surface is exposed again, and the wettability is strongly reduced. The lateral sample diffusion outside the wells is therefore limited and results in sample up-concentration within the wells, as shown in Figure 3D. This procedure presumably also reduces cross contamination during recrystallization, which is crucial to maintain the isolated nature of the droplets within the array.

### The Effect of Recrystallization on Signal Intensity

**Figure 4:**
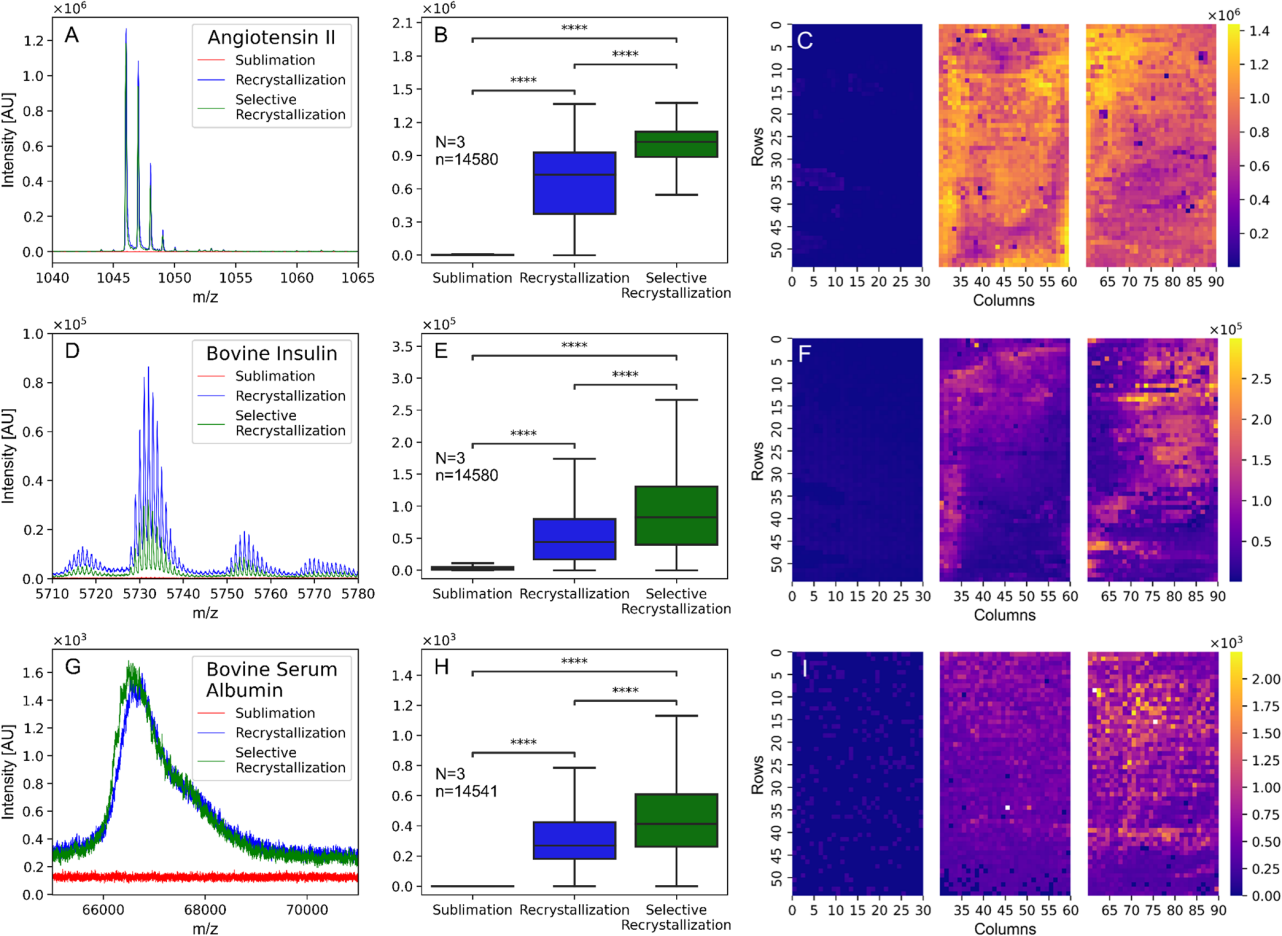
Dependency of the analyte signal intensity relative to the matrix delivery method. One condition was tested per droplet array block on the same plate in replicates of 3. [A–C] Representative MALDI mass spectra of 20 μM angiotensin II, bovine insulin, and bovine serum albumin in positive mode. [D–F] Absolute signal intensities of the model proteins were obtained with different sample preparation methods. [G–I] Heatmaps of an entire droplet array coated with 20 μM angiotensin II, bovine insulin, and bovine serum albumin after sample preparation. Each pixel represents the normalized intensity of one individual well. Statistical replicates annotated in each graph.

In the next step, we compared the signal intensities of three model proteins with the three different preparation methods. The absolute intensities of angiotensin II at *m*/*z* 1046.54 [M + H]^+^, insulin at *m*/*z* 5733.49 [M + H]^+^, and bovine serum albumin at *m*/*z* 66620 [M + H]^+^ were taken as readout (Figure 4A–C). Here, we used a droplet array with three separate blocks of 1620 wells, which were filled with the same analyte solution containing 20 μM of each protein. After matrix sublimation, each block was subsequently employed for one method (sublimation, recrystallization, and selective recrystallization). Therefore, small variations in sample and matrix contents between experiments could be minimized. We confirmed that repeated introduction into the instrument chamber and exposure to the high vacuum during the measurements had no influence on the result, i.e., the matrix compound did not evaporate within the time frame of the measurement (Figure S6). As shown in Figure 4, we detected low signals for angiotensin II and insulin in the sublimated matrix without recrystallization, and it was not possible to record a signal of BSA. However, for all three proteins, we found a significant increase in signal when a recrystallization step was done before the measurements. The heatmaps in Figure 4C,F,I show this significant increase all over the droplet array, and they also indicate a good homogeneous signal intensity over the whole plate. Next, the matrix was selectively removed around the wells before the recrystallization was conducted. During recrystallization, both matrix and sample can mix homogeneously in the liquid state. This leads to a better energy transfer in the matrix and, therefore, a better ionization efficiency, i.e., higher signals. Again, the heatmaps underline the good homogeneous distribution across the entire droplet array.

In summary, it could be shown that recrystallization of the sublimated matrix yields an improved ionization efficiency and a more homogeneous substrate layer leading to higher MALDI-MS signal intensities across each of our chosen analytes. Additionally, by removing the matrix surrounding the wells before recrystallization, this effect was significantly enhanced. This resulted in an average signal intensity increase of 50.71% for angiotensin II, 73.12% for bovine insulin, and 48.12% for bovine serum albumin.

## Conclusions

Sample preparation is crucial to the success of MALDI-MS measurements. This is particularly important when the number of measurements is drastically increased as in the case of droplet arrays where the preparation should be fast, facile, and in parallel for all sample droplets. Traditional matrix deposition methods such as pipetting and matrix spraying are not well-suited for such high-throughput applications. Here, we compared different methods to apply matrix compounds and obtain matrix-analyte crystals. We achieved significant improvements in signal intensity as well as spatial resolution by implementing a workflow of matrix sublimation, removal of excess matrix around the wells, and recrystallization. Given the ever-increasing importance of high-throughput analytical methods, including mass spectrometry, we have shown a strategy to obtain high signals with little additional effort in the sample preparation steps. The method for sample preparation can be straightforwardly employed on arrays with higher droplet density, where the plate carries even more droplets (up to 100 000) and droplet size is further downscaled to subnanoliter volumes. This approach overcomes the limitations in throughput of standard MALDI target plates with 384 sample positions and allows for new endeavors in the label-free analysis of pico-/nanoliter droplets, as an alternative or as an orthogonal method to optical methods including fluorescence spectroscopy. Applications include drug and biomarker discovery, monitoring enzymatic assays, protein engineering, single-cell analysis, and combinatorial chemistry.

## Supporting Information Available

The custom-made sublimation chamber (Figure S1), MALDI MS heatmaps of three plates filled with bovine insulin (Figure S2), distribution of the fluorescence intensity of a selected area of the plate (Figure S3), selected spotting of droplets and assessment of cross talk and wetting process between wells (Figures S4 and S5), stability of 2.5 DHB assessed with repeated measurements on the same plate (Figure S6) (PDF)
