# Supplementary material for: MALDI Mass Spectrometry on High-Density Droplet Arrays: Matrix Deposition, Selective Removal, and Recrystallization
Source: ACS Meas Sci Au. 2024 Jul 5;4(5):488–95. doi: 10.1021/acsmeasuresciau.4c00016 (PMC11487676; doi:10.1021/acsmeasuresciau.4c00016)
Supplement: Supplementary file 1 — tg4c00016_si_001.pdf [file tg4c00016_si_001.pdf]

MALDI Mass spectrometry on high-density droplet arrays: Matrix deposition, selective removal, and recrystallization

Simon F. Berlanda, Maximilian Breitfeld and Petra S. Dittrich*

Supplementary Information

ETH Zurich, Department of Biosystems Science and Engineering, Schanzenstrasse 44, CH-4056 Basel, Switzerland

* Corresponding Author

* Email: petra.dittrich@bsse.ethz.ch

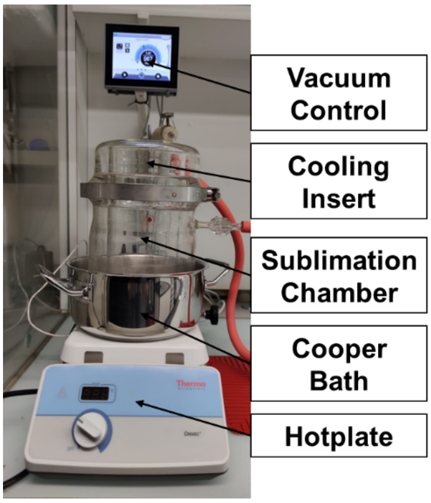

Figure S1. Custom-made sublimation chamber for matrix deposition on droplet arrays.

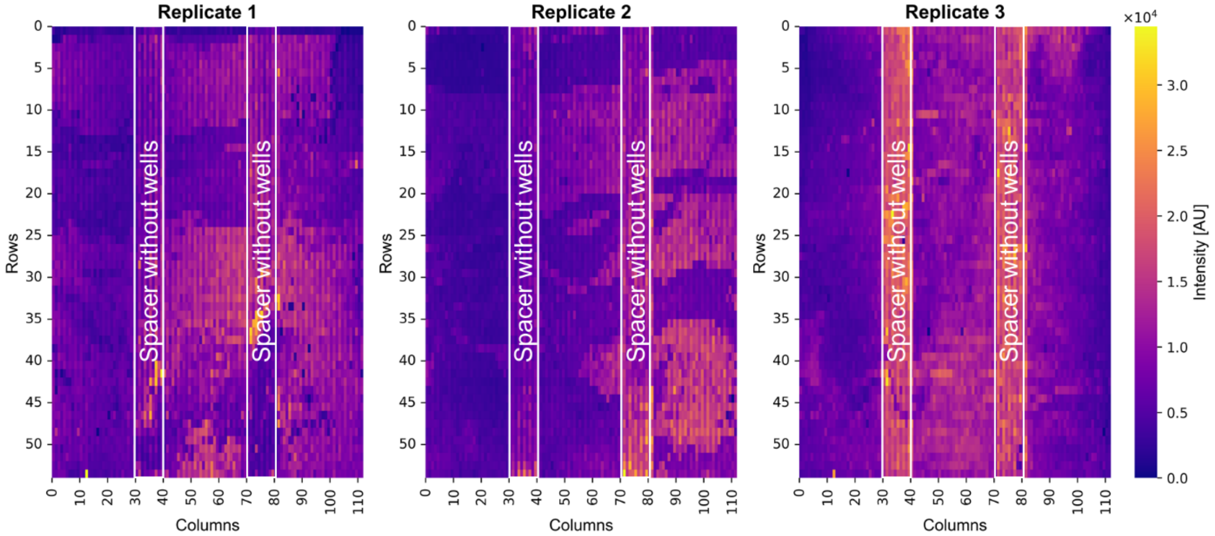

Figure S2. MALDI MS heatmaps of three plates to show the complete filling of the plate and the plate-to-plate reproducibility. The MALDI-MS heatmaps are taken on three array plates filled with 40 µM bovine insulin. Each plate consists of three blocks of wells separated by spacer regions, in total the plate has 4950 sample wells. MALDI-MS imaging was carried out with a custom target geometry including the spacer regions without wells in the same pattern as the array. The large spacers of SU-8 coating still retains analyte, while the patterned surface of the array region allows for encapsulation of sample. Each pixel represents the normalized intensity of bovine insulin in one individual well.

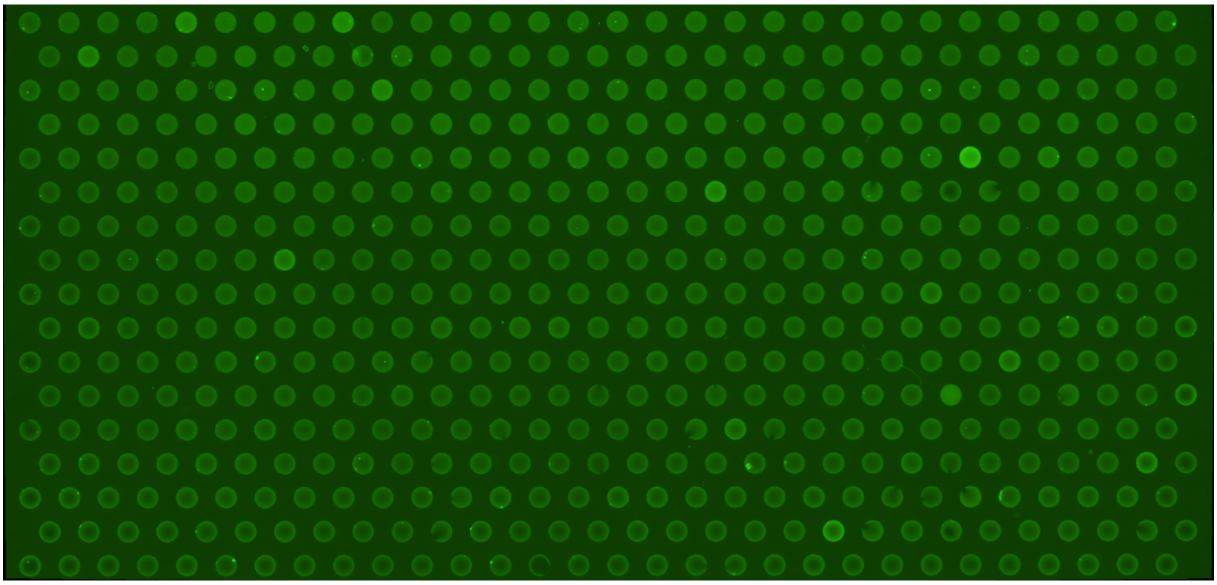

Figure S3. Distribution of the fluorescence intensity of 40 µM insulin-FITC over the array plate. Fluorescence imaging was carried out on a Nikon Eclipse Ti2 in the excitation wavelength region between 446 and 486 nm and emission at 500 to 550 nm.

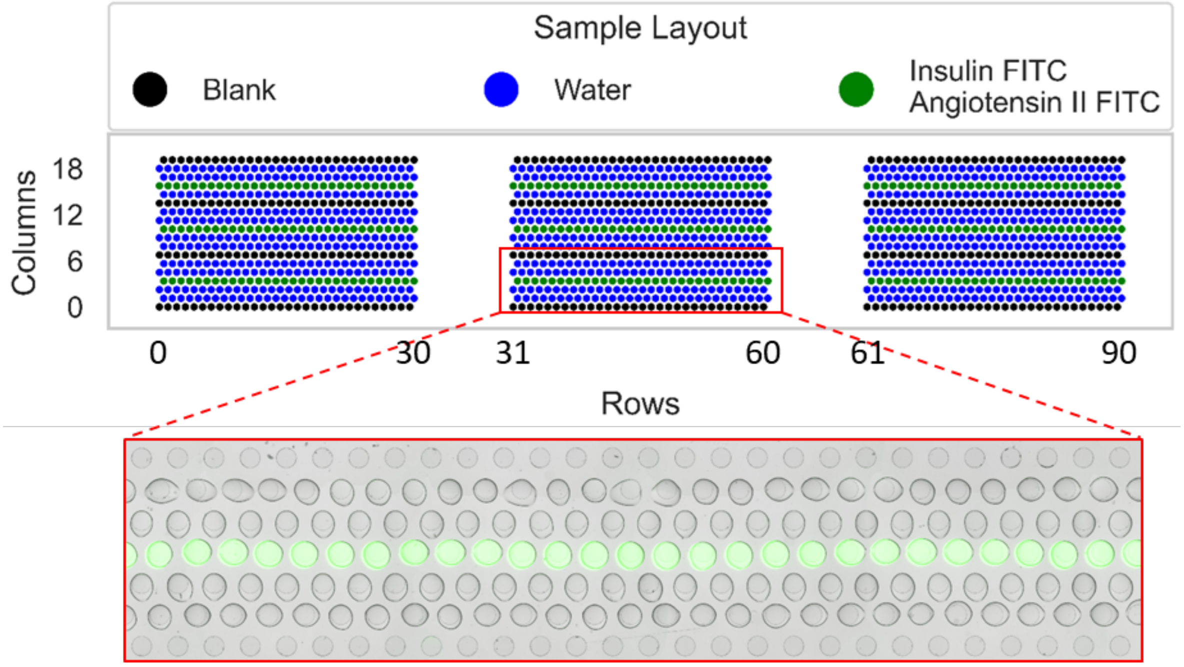

Figure S3. Droplet array generation and deposition via droplet spotting. Top: Sample layout for the open droplet arrays on ITO coated plates (not to scale). Bottom: Micrograph of a droplet array containing a mix of 15 µM insulin-FITC and angiotensin II-FAM, as well as water blanks and empty rows. (n = 1710 spots)

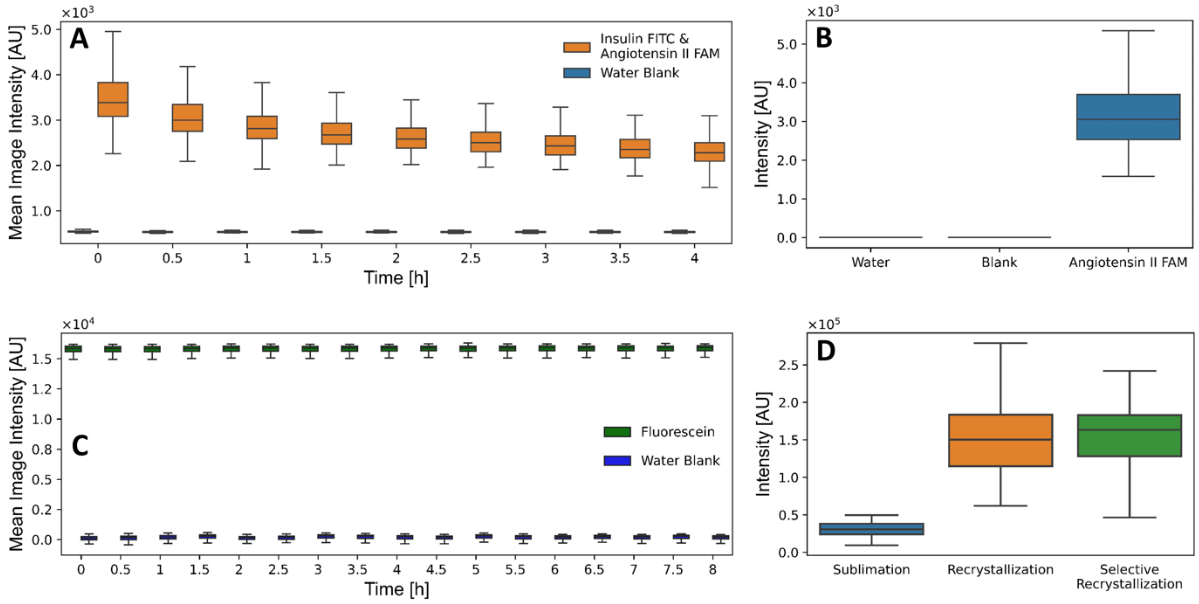

Figure S4. Assessment of cross talk and wetting process between wells. Fluorescence images were background-corrected through subtraction of the empty rows and MALDI-MS spectra were recorded as described in the main manuscript, but corrected to a mass range of 900-2000 Da. [A] Time-lapsed fluorescence imaging of a spotted array plate filled with alternating rows of 15 µM insulin-FITC & angiotensin II-FAM, water, and blanks. The intensity of water did not change over time indicating no cross talk between individual wells. [B] Absolute signal intensities of angiotensin II-FAM taken at m/z 1405 for each well, after sublimation of matrix over the whole plate. [C] Time-lapsed fluorescence imaging of a spotted array plate with 20 µM fluorescein and reduced illumination intensity to avoid bleaching over 8h. [D] The dependency of analyte signal intensity for angiotensin II-FAM on the matrix application method was assessed. Each sample preparation condition was tested on the same plate by dividing the plate in three blocks (n = 1710 spots).

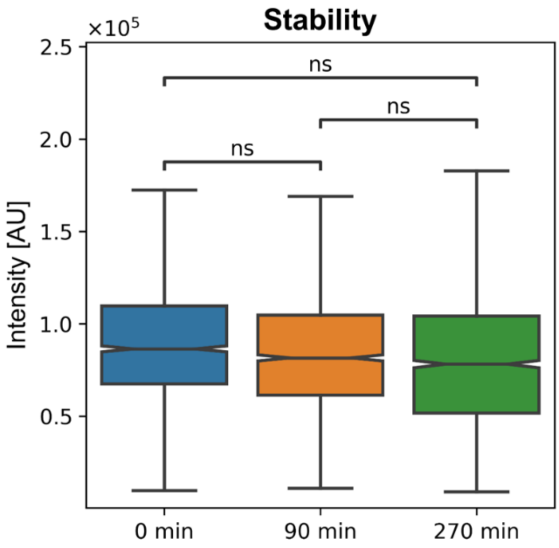

Figure S6. Stability assessment. A plate with 6048 wells is filled with 40 µM bovine insulin and the matrix 2,5-DHB is added by sublimation. MALDI mass spectra are recorded for one third (=2016 wells) of the plate, the other areas are measured after 90 and 270 minutes, respectively. The average intensity remains the same, confirming that the fully prepared plate with analyte and matrix can be used and exposed to the high vacuum of the instrument multiple times without loss of analyte or matrix.
